# Barriers and challenges in integration of anthroposophic medicine in supportive breast cancer care

**DOI:** 10.1186/2193-1801-2-364

**Published:** 2013-07-31

**Authors:** Eran Ben-Arye, Elad Schiff, Moti Levy, Orit Gressel Raz, Yael Barak, Gil Bar-Sela

**Affiliations:** Integrative Oncology Program, Oncology Service, Lin Medical Center, Clalit Health Services, Haifa and Western Galilee District, 35 Rothschild St, Haifa, 35152 Israel; Department of Family Medicine, Faculty of Medicine, Complementary and Traditional Medicine Unit, Technion-Israel Institute of Technology, Haifa, Israel; Department of Internal Medicine and Integrative Surgery Service, B’nai Zion Hospital, Haifa, Israel; The International Center for Health, Law, and Ethics, Haifa, Israel; Clalit Complementary Medicine, Clalit Health Services, Haifa, Israel; Division of Oncology, Rambam Health Care Campus, Haifa, Israel

**Keywords:** Integrative medicine, Anthroposophic medicine, Viscum album, Quality of life, Complementary medicine, Cancer

## Abstract

In the last decade, more and more oncology centers are challenged with complementary medicine (CM) integration within supportive breast cancer care. Quality of life (QOL) improvement and attenuation of oncology treatment side effects are the core objectives of integrative CM programs in cancer care. Yet, limited research is available on the use of specific CM modalities in an integrative setting and on cancer patients’ compliance with CM consultation. Studies are especially warranted to view the clinical application of researched CM modalities, such as anthroposophic medicine (AM), a unique CM modality oriented to cancer supportive care. Our objective was to characterize consultation patterns provided by physicians trained in CM following oncology health-care practitioners’ referral of patients receiving chemotherapy. We aimed to identify characteristics of patients who consulted with AM and to explore patients’ compliance to AM treatment. Of the 341 patients consulted with integrative physicians, 138 were diagnosed with breast cancer. Following integrative physician consultation, 56 patients were advised about AM treatment and 285 about other CM modalities. Logistic multivariate regression model found that, compared with patients receiving non-anthroposophic CM, the AM group had significantly greater rates of previous CM use [EXP(B) = 3.25, 95% C.I. 1.64-6.29, p = 0.001] and higher rates of cancer recurrence at baseline (p = 0.038). Most AM users (71.4%) used a single AM modality, such as mistletoe (viscum album) injections, oral AM supplements, or music therapy. Compliance with AM modalities following physician recommendation ranged from 44% to 71% of patients. We conclude that AM treatment provided within the integrative oncology setting is feasible based on compliance assessment. Other studies are warranted to explore the effectiveness of AM in improving patients’ QOL during chemotherapy.

## Introduction

Integrating complementary medicine (CM) within cancer supportive care is an increasing phenomenon in conventional cancer institutions across the globe (Ben-Arye et al. [2013]). Anthroposophic medicine (AM) was one of the first CM modalities integrated within conventional oncology care settings and is mostly well-known due to the extensively studied plant mistletoe (V*iscum album*) in *in vitro* and clinical studies (Kienle [2006]; Horneber et al. [2008]; Lev et al. [2011]). In a systematic review, Kienle et al. found that quality of life (QOL) and tolerability of chemotherapy, radiotherapy or surgery were improved in patients receiving mistletoe in 21 of 24 clinical studies (15 randomized, 9 non-randomized) conducted with breast and gynecological cancer patients (Kienle et al. [2009]). In the arena of basic science breast cancer research, mistletoe effects included prevention of surgery-induced suppression of granulocyte function (Büssing et al. [2005]), DNA repair in damaged peripheral blood mononuclear cells (Kovacs [2002]), cytotoxic effect (Martín-Cordero et al. [2001]), and effects in different breast cancer cell lines on immune defense and stress response genes, as well as on cell-cell adhesion and cytoskeleton pathways (Eggenschwiler et al. [2006]).

Anthroposophic medical care is based on collaborative teamwork of physicians, nurses, art therapists, physical/massage therapists, psychotherapy and biography counseling, and more. AM practitioners perceive spiritual elements in the healing process and target treatment to strengthen the organism by stimulation of self-healing (Kienle et al. [2006]). AM modalities in cancer care may include nutritional, herbal (including mistletoe treatment), homeopathic and herbal remedies as well as referral to anthroposophic practitioners (e.g. music therapists).

In this study, we explore the use of anthroposophic medicine in a setting where AM is provided as part of an Integrative Oncology Program (IOP) operating within a conventional oncology service of the largest health maintenance organization in northern Israel. The inclusion of the IOP within the oncology service supportive care was launched in 2008, aiming at improving patients’ quality of life during chemotherapy and advanced disease states (Ben-Arye et al. [2012a]). The study of AM provided in this setting is unique since referral to CM consultation is determined by oncology health care providers (HCPs include oncologists, nurses, social workers) on a free-of-charge basis while outcomes are monitored using a research-based registry protocol. Our study objectives were to identify characteristics of patients receiving AM compared to non-AM complementary therapies, to explore clinical indications for the use of AM-based treatments, and to evaluate compliance with AM treatments in this setting.

## Materials and methods

### Study sites and participants

The study took place at two oncology centers operated by Clalit Health Services in northern Israel between July 2009 and January 2013. These oncology services provide conventional oncology treatment to more than 1000 new patients each year.

### Registry protocol data collection

The clinical activities of the integrative oncology program (IOP) are documented in a research-based registry protocol. Referral to the IOP may be initiated by the patient’s oncologist, oncology nurse or social worker, and is limited to patients treated within the oncology service during chemotherapy and/or advanced cancer. Following the referral, an initial integrative medical intake interview was scheduled with an integrative physician (IP) who is a medical doctor with extensive training in the field of CM (including AM). The IP interview lasted approximately an hour, and addressed the patient’s past experience and present expectations regarding the use of CM. Each session typically concluded with an outlining of treatment goals, followed by the construction of a preliminary treatment plan tailored to the patient’s outlook and level of evidence (efficacy, safety, possible interactions with chemotherapy, etc.). The CM treatment plan was provided by the IPs or CM practitioners and may have included herbal/dietary supplements and nutritional consultation; acupuncture and manual modalities; anthroposophic medicine; and mind-body therapies (e.g. guided imagery, music therapy, and spiritual counseling). The frequency of CM treatments ranged from once a week to once every three weeks. Following 2–4 months of treatment, a concluding clinical assessment was performed.

### Data analysis

Data were evaluated using the SPSS software program (version 18; SPSS Inc., Chicago, IL). Pearson’s chi-square test and Fisher’s exact test were used to detect differences in the prevalence of categorical variables and demographic data between the participants in various groups. In addition, a *t*-test was performed to determine any differences in the continuous variables when normality was assumed. In cases of non-normal distribution, the Mann–Whitney *U* test was used. A logistic multivariate regression model was developed to assess the factors associated with patients receiving AM treatments versus those receiving other CM modalities. The regression model included patients’ age, gender, language (identifying Hebrew, Arabic and Russian-speaking participants), use of CM for cancer-related outcomes or for general non-cancer-related reasons, and prevalence of cancer recurrence. A p value of ≤0.05 was considered to be of statistical significance.

### Ethical considerations

Prior to initiation of the study, approval was received from the Ethics (Helsinki) Committee at the Carmel Medical Center, Haifa, Israel. Participation in the study was voluntary, and information was collated in an anonymous fashion, after the patient signed an informed consent.

## Results and discussion

During the study period, 341 patients received consultation by the IP following an HCP referral. All the participating patients were undergoing either active chemotherapy, palliative treatment, or both. Table 1 compares demographics, treatments, and cancer-related variables between patients who consulted to receive an AM-based CM treatment regimen (AM group; n = 56, 16.4%) and those who consulted with no AM CM treatment regimen (non-AM group; n = 285, 83.6%). The most frequent cancers were breast (138 patients, 41.8%), gastrointestinal (78, 23.6%), and gynecological (65, 19.7%).

**Table 1:** **Comparison of demographic, treatment, and cancer-related variables between AM- recommended treatment regimen patients and non-AM recommended treatment regimen patients**

Characteristic	Total cohort N = 341, n (%)	AM group N = 56, n (%)	Non-AM group N = 285, n (%)	***P*** value
**Age** [Mean in years ± SD (median)]	62.26 ± 12.58 (63)	64.05 ± 11.55 (64.5)	61.91 ± 12.77 (63)	0.24
**Sex**				
Male	84 (24.6)	12 (21.4)	72 (25.3)	0.63
Female	257 (75.4)	44 (78.6)	213 (74.7)	
Main language*:				
Hebrew	243 (73.4)	47 (85.5)	196 (71.0)	Hebrew vs. non-Hebrew
Arabic	32 (9.7)	3 (5.5)	29 (10.50)	
Russian	56 (16.9)	5 (9.1)		P = 0.029
Country of birth*				
Israel	157 (48.2)	28 (52.8)	129 (47.3)	Israeli-born vs. others
Europe/America	84 (25.8)	13 (24.5)	71 (26.0)	
Asia/Africa	30 (9.2)	6 (11.3)	24 (8.8)	P = 0.55
Former USSR	55 (16.9)	6 (11.3)	49 (17.9)	
Residence distance*				
Haifa**	134 (39.3)	21 (37.5)	113 (39.6)	0.88
Suburbs**	130 (38.1)	21 (37.5)	109 (38.2)	
Periphery**	77 (22.6)	14 (25.0)	63 (22.1)	
Cancer sites*				
Breast	138 (41.8)	24 (44.4%)	114 (41.3%)	0.76
Gynecological	65 (19.7%)	13 (24.1%)	52 (18.8%)	0.36
Gastrointestinal	78 (23.6%)	10 (18.5%)	68 (24.6%)	1.00
Prostate + urologic	31 (9.4%)	5 (9.3%)	26 (9.4%)	0.38
Lung	18 (5.5%)	2 (3.7%)	16 (5.8%)	0.74
Cancer recurrence*				
Recurrent	82 (24.0)	19 (33.9)	63 (22.1)	0.062
Non-recurrent	259 (76.0)	37 (66.1)	222 (77.9)	
Evidence for advanced cancer*				
Metastases	159 (46.6)	28 (50.0)	131 (46.0)	0.66
Non-metastatic	182 (53.4)	28 (50.0)	154 (54.0)	
Chemotherapy setting*				
Neoadjuvant + adjuvant	209 (65.7)	34 (61.8)	175 (66.5)	0.53
Palliative + Curative	109 (34.3)	21 (38.2)	88 (33.5)	
Non-cancer related CM use*				
Users	165 (48.5)	42 (75.0)	123 (43.3)	<0.0001
Non-users	175 (51.5)	14 (25.0)	161 (56.7)	
Cancer-related CM use*				
Users	170 (50.1)	39 (69.9)	131 (46.3)	0.002
Non-users	169 (49.9)	17 (30.4)	152 (53.7)	

Notes:

*SD* standard deviation; Data analysis was performed by *t*-test, Fisher’s exact test, and Pearson Chi-square test.

*Data is limited to the number of respondents who reported this information.

**In relation to residential distance from Haifa: suburbs – up to 20 km from Haifa; periphery –beyond 20 km from Haifa.

There were no significant differences between the two groups with respect to age, gender, country of birth, distance of residence from the oncology center, extent of cancer involvement (local vs. metastatic disease), recurrence, and chemotherapy setting (e.g., neo-adjuvant and adjuvant vs. palliative). Nevertheless, patients receiving AM were predominantly Hebrew speakers (p = 0.014, compared with Arabic and Russian) and used CM significantly more for general (p < 0.0001) or cancer-related outcomes (p = 0.002) prior to the IP consultation.

### Factors predicting anthroposophic medicine consultation

A logistic multivariate regression model was conducted to assess the independency of the above variables. An AM-based treatment regimen was associated with previous CM use [EXP(B) = 3.25, 95% C.I. 1.64-6.29, p = 0.001], CM use for cancer-related outcomes [EXP(B) = 2.34, 95% C.I. 1.21-4.51, p = 0.011], cancer recurrence [EXP(B) = 2.03, 95% C.I. 1.03-3.98, p = 0.038], and Hebrew speakers [EXP(B) = 2.96, 95% C.I. 1.28-6.79, p = 0.011].

### Treatment goals of AM and non-AM treatment regimens

**Figure 1:**
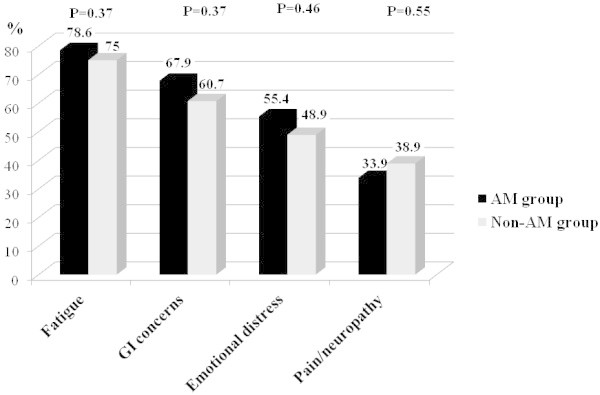
**Integrative treatment goals (% of patients in each group).**

Figure 1 shows that integrative physicians had similar treatment goals for patients recommended to AM or non-AM treatments. Leading treatment goals were (in decreasing order) fatigue improvement (75–78.6% of patients), amelioration of gastrointestinal concerns (60.7-67.9%; e.g., stomatitis, nausea, constipation, diarrhea, weight & appetite change), alleviation of emotional distress (48.9-55.4%), and pain and neuropathy management (33.9-38.9%).

### AM modalities use: clinical indications and compliance

In Table 2, we present three AM medicine modalities prescribed for quality of life (QOL) improvement: Mistletoe (*Viscum album*) injections, AM supplements, and AM music therapy. Forty of the 56 AM users (71.4%) used only one of these three modalities and 16 (28.6%) used combined AM therapies. Common clinical indications for AM use and specific AM supplements used are specified in Table 2.

**Table 2:** **Clinical indications of 3 anthroposophic medicine modalities prescribed for quality of life (QOL) improvement**

AM modality	Number of AM users	Common QOL-oriented clinical indications (including specific ***supplements indicated***)	Patient compliance
	n (%)		n (%)
**Mistletoe injections**	23 (41.1)	Fatigue	12 (52.2)*
		Leukopenia	
		Chemotherapy-induced	
		neuropathy	
		Pain	
**AM supplements**	28 (50.0)	Fatigue (*Levico D1 or Levico comp D3*)	20 (71.4)*
		Chemotherapy-induced	
		neuropathy (*Aconite nervenoil*)	
		Insomnia (*Soleum uleginosum; Avena sativa comp, Hepatodoron*)	
**AM music therapy**	25 (44.6)	Anxiety, restlessness Insomnia	11 (44)**
		Chemotherapy-induced	14 (56)***
		neuropathy	
		Dyspnea	
		End-of-life care	
		Pain relief	
		Nausea, constipation	
		Fatigue	

Notes:

* Documented use in medical file.

** Up to 3 sessions.

***More than 3 sessions.

Compliance with AM treatments was assessed by comparing IP recommendations in the initial visit to the report in the concluding clinical assessment following 2–4 months of treatment. Of the three AM modalities, AM supplement use had the highest patient compliance rate (71.4%) followed by mistletoe injections (52.2%) and optimal music therapy sequence of more than three sessions (44%). Overall, 40 of the 56 patients in the AM group complied with at least one of the three AM therapies (71.4%).

## Discussion

This study focuses on the characteristics of AM use provided within a unique setting of CM consultation and treatment integrated within a conventional public oncology service in the largest HMO in Israel. Although CM use by people with cancer in Israel is highly prevalent (about 50% of patients during chemotherapy) (Ben-Arye et al. [2012b]; Ben-Arye et al. [2006]), the use of AM is limited by the following potential reasons: lack of AM coverage in the Israeli national medical insurance plans, unfamiliarity with AM philosophy and practice, reluctance to inject mistletoe subcutaneously, and limited accessibility to physicians and non-physician practitioners who practice AM. In our study, some of these potential limitations were reduced due to a built-in referral process by an IP and practitioners who have received extensive training in AM. The construction of the integrative treatment plan is patient-centered and determined by a variety of factors: a) the spectrum of patients’ concerns, symptoms, and well-being; b) level of research supporting CM modality efficacy and safety (including interactions with chemotherapy) in specific oncology settings (cancer site, stage, and treatment protocol); c) patient readiness to experience different CM modalities. This patient-centered design may be considered as a major bias in determining referral to AM rather than non-AM integrative treatments. Nevertheless, our methodology research of a non-randomized preference study tailored to patients’ needs and concerns can be also perceived as a real-world pragmatic trial.

One of the major findings in our study is that patients in the AM group had higher rates of cancer recurrence compared with patients receiving non-AM CM consultation. This finding may suggest that AM may be considered in our setting as a second-line of integrative treatment compared with other CM modalities, such as acupuncture, herbs and nutrition, that may be perceived as more familiar or less invasive. Other factors significantly associated with AM use, like previous CM use and the predominance of Hebrew speakers, emphasize the need to conduct more studies on the potential bias of AM inclusion in integrative medicine treatment regimens recommended by IPs. Specifically, qualitative studies are warranted to detect patient- as well as IP-associated factors that may influence AM-oriented consultations. Another point to consider in future studies is whether the paucity of AM in integrative treatment plans is also determined by quality-of-life indications and the potential of AM to improve chemotherapy side effects and patients’ symptoms. In our study, we found no statistically significant difference in IP treatment goals between the AM and non-AM groups regarding four leading concerns: fatigue, gastrointestinal symptoms, emotional distress, and pain/neuropathy. This distribution of treatment goals may also be dependent on the status of rigorous research on the efficacy and safety of AM in cancer supportive care. Most mistletoe treatment for cancer studies are in the *in vitro* setting (Mengs et al. [2002]) or focus on survival (Ostermann et al. [2009]) rather than QOL aspects which are more relevant in an integrative oncology setting. Currently, the spectrum of published mistletoe QOL-oriented research is limited to studies assessing general well-being improvement (Brandenberger et al. [2012]; Eisenbraun et al. [2011]; Kienle & Kiene [2010]; Semiglazov et al. [2006]; Piao et al. [2004]). Studies on specific symptom improvement include leukopenia and diarrhea in gastric cancer patients (Kim et al. [2012]), reducing malignant ascites (Bar-Sela et al. [2006]), improving emotional concerns in palliative care (Heusser et al. [2006]), and lessening of nausea/vomiting in breast cancer patients receiving adjuvant chemotherapy (Loewe-Mesch et al. [2008]). Most importantly, mistletoe safety aspects have also been studied and complement the emerging data on efficacy (Kienle et al. [2011]; Augustin et al. [2005]). In contrast to this growing body of research on mistletoe efficacy and safety, there is limited research regarding other AM supplements and art therapies that may enhance patients’ supportive cancer care. Encouraging data on the impact of art therapies on the QOL of cancer patients was reported in studies with disease-free breast cancer patients treated with multimodal therapy that included eurythmy and painting therapy (improved sleep and cancer-related fatigue) (Kröz et al. [2012]), and in patients during chemotherapy participating in art therapy sessions (Bar-Sela et al. [2007]). As research develops in clinical practice, we suggest that the referral of patients to AM will also grow and enable IPs to better recommend AM, based on research evidence.

In this manuscript, we do not report on the clinical outcomes of patients in the AM and non-AM groups. Data on these aspects will be published elsewhere. Our intention was to focus on AM consultation patterns and treatments within an integrative oncology setting rather than on outcome data. In this regard, the report on compliance to AM modalities ranging between 44% to 71% of patients should be carefully interpreted. In most studies on compliance aspects in cancer care, researchers examined the association between patients’ compliance with standard treatment and CM used (Söllner et al. [2000]). To the best of our knowledge, our study is the first report on CM compliance in an integrative oncology setting. Although more studies are warranted on CM and cancer patients’ compliance, preliminary insight can be achieved through assessment of cancer patient’s compliance to medication use. Studies regarding non-compliance and adherence of patients with cancer to drug therapies associate non-compliance with drug side effects (letrozole in breast cancer, 18.4% non-compliance rate) (Fontein et al. [2012]), forgetting to take medications, side effects and misunderstanding instructions (capecitabine, 9%) (Winterhalder et al. [2011]). More studies are needed to explore the reasons for non-compliance or lack of adherence in patients receiving AM in integrative settings. Potential motives for AM treatment initiation and continuation versus non-compliance may encompass various domains, such as symptom severity, clinical improvement or deterioration that may be related to AM specific effects or to a more non-specific therapeutic alliance with the AM practitioner, cost considerations, and more.

Our study has potential limitations and its results should be cautiously interpreted or generalized to other settings of care. As stated previously, the major limitation concerns with the non-controlled preference study methodology. The pragmatic trial of patient-centered tailoring of a treatment plan may have caused selection bias of patients who were more compliant with AM or have more advanced disease. Other limitations include the culturally diverse mosaic of the cancer patient populations in north Israel that may affect patients’ affinity towards AM due to historical and religious motives (e.g., religious Jewish and Muslim patients’ reluctance of an apparently spiritual modality that may be affiliated with Christianity, averseness of Holocaust survivors regarding a Central European-dominant modality or attractiveness of secular Israeli-born and immigrant patients to apparently non-religious spirituality). Another study limitation is the accessibility of patients to receive AM treatments in two aspects: a) geographical – long residence distance from the cancer center may have hindered AM provision (music therapy in particular); b) financial – although IP consultations and music therapy were provided at no cost, mistletoe injections and other AM supplements were purchased on an out-of-pocket basis and thus cost considerations may have influenced treatment compliance and adherence. Last but not least, we did not evaluate the reasons for non-compliance and further studies are needed to explore these aspects.

## Conclusions

Anthroposophic medicine is an important modality that can foster supportive cancer care, especially in patients with breast cancer receiving chemotherapy. Leading clinical indications for AM referral and treatment include fatigue, gastrointestinal concerns, emotional concerns, pain and neuropathy. Assessing patient compliance and adherence to AM referral and treatment should be regarded as an important monitoring procedure that advances the learning process and attentiveness of health care providers to the barriers and challenges of optimal integrative treatment tailoring.
